# The genetic association of *RUNX3* with ankylosing spondylitis can be explained by allele-specific effects on IRF4 recruitment that alter gene expression

**DOI:** 10.1136/annrheumdis-2015-207490

**Published:** 2015-10-09

**Authors:** Matteo Vecellio, Amity R Roberts, Carla J Cohen, Adrian Cortes, Julian C Knight, Paul Bowness, B Paul Wordsworth

**Affiliations:** 1 Nuffield Department of Orthopaedics, Rheumatology and Musculoskeletal Sciences, University of Oxford, Oxford, UK; 2 National Institute for Health Research Oxford Musculoskeletal Biomedical Research Unit, Oxford, UK; 3 National Institute for Health Research Oxford Comprehensive Biomedical Research Centre, Botnar Research Centre, Nuffield Orthopaedic Centre, Oxford, UK; 4 Division of Clinical Neurology, Nuffield Department of Clinical Neurosciences, John Radcliffe Hospital, University of Oxford, Oxford, UK; 5 Wellcome Trust Centre for Human Genetics, University of Oxford, Oxford, UK

**Keywords:** Ankylosing Spondylitis, T Cells, Gene Polymorphism, Spondyloarthritis

## Abstract

**Objectives:**

To identify the functional basis for the genetic association of single nucleotide polymorphisms (SNP), upstream of the *RUNX3* promoter, with ankylosing spondylitis (AS).

**Methods:**

We performed conditional analysis of genetic association data and used ENCODE data on chromatin remodelling and transcription factor (TF) binding sites to identify the primary AS-associated regulatory SNP in the *RUNX3* region. The functional effects of this SNP were tested in luciferase reporter assays. Its effects on TF binding were investigated by electrophoretic mobility gel shift assays and chromatin immunoprecipitation. *RUNX3* mRNA levels were compared in primary CD8+ T cells of AS risk and protective genotypes by real-time PCR.

**Results:**

The association of the *RUNX3* SNP *rs4648889* with AS (p<7.6×10^−14^) was robust to conditioning on all other SNPs in this region. We identified a 2 kb putative regulatory element, upstream of *RUNX3*, containing *rs4648889.* In reporter gene constructs, the protective *rs4648889* ‘G’ allele increased luciferase activity ninefold but significantly less activity (4.3-fold) was seen with the AS risk ‘A’ allele (p≤0.01). The binding of Jurkat or CD8+ T-cell nuclear extracts to the risk allele was decreased and IRF4 recruitment was reduced. The AS-risk allele also affected H3K4Me1 histone methylation and associated with an allele-specific reduction in *RUNX3* mRNA (p<0.05).

**Conclusion:**

We identified a regulatory region upstream of *RUNX3* that is modulated by *rs4648889*. The risk allele decreases TF binding (including IRF4) and reduces reporter activity and *RUNX3* expression. These findings may have important implications for understanding the role of T cells and other immune cells in AS.

## Introduction

Ankylosing spondylitis (AS) is a common form of spondyloarthropathy (SpA) with numerous robust genetic associations, of which most are currently unexplained. Some functional protein-coding single nucleotide polymorphisms (SNPs) have been identified in genes associated with AS, such as *HLA-B*, *ERAP1* and *IL23R*.^1–4^ More commonly, the associated SNPs lie in non-coding flanking sequences or intragenic regions where they may influence gene expression.^1^ ^2^ ^5^ Genome-wide association studies (GWAS) show convincing association between *RUNX3* and AS and psoriatic arthritis, another form of SpA.^1^ ^2^ ^6^ This association extends to a cluster of SNPs at the *RUNX3* locus,^1^ located from 0.5 to 2 kb upstream of the *RUNX3* promoter. One of these, *rs4648889*, lies in a putative transcription regulatory region defined by DNaseI hypersensitivity and the presence of chromatin marks correlated with active enhancers (H3K4Me1 and H3K27Ac).^7^ *RUNX3* is a member of the runt domain-containing family of transcription factors (TFs), which play key roles in many developmental pathways.^8^ The *Runx3*^−/−^ mouse shows a complex phenotype affecting several organs, highlighting the broad spectrum of *RUNX3* action.^9^ *RUNX3* influences several types of immune cell that could be involved in AS, including natural killer (NK) cells and regulatory T cells,^10^ ^11^ but seems particularly involved in the differentiation and development of CD8+ T cells.^12^ ^13^ It seems relevant that CD8+ T-cell numbers are reduced in AS and that this is related to the *RUNX3* genotype.^1^ ^2^ There is evidence for epigenetic regulation of *RUNX3* expression; the gene has two differentially expressed alternative promoters resulting in proteins that have dissimilar transcriptional activation capacity.^14^ ^15^ The distal promoter is specifically demethylated in T cells^16^ but is methylated on both alleles in clonal cell populations.^17^ *RUNX3* promoter methylation status has also been incriminated in acute myeloid leukaemia^18^ and malignant transformation of ovarian endometriosis.^19^

Numerous SNPs in a ∼15 kb linkage disequilibrium (LD) block upstream of *RUNX3* are strongly associated with AS. In the recent large ImmunoChip study, the lead SNP was *rs6600247* (p=1.3×10^−14^), which is in complete LD with *rs4648889* (∼2 kb upstream of the *RUNX3* promoter).^1^ Here we apply conditional analysis to investigate the LD patterns within this region to identify the SNP(s) most likely to be primarily associated with AS. We also use historic ENCODE data on the transcriptional and epigenetic regulation of *RUNX3*,^20^ together with in vitro and ex vivo molecular assays to investigate functional effects of *rs4648889* on *RUNX3* expression.

## Methods

### Genotyping

Historical typing data from the AS ImmunoChip study^1^ were available for some patients or were otherwise obtained using TaqMan SNP assay (Life Technologies, Paisley, UK) to assign *rs4648889* genotypes. Where required, DNA was extracted using the Qiagen AllPrep DNA/RNA Mini Kit (Qiagen, Manchester, UK).

### Imputation

Genotype data from the UK cases in the International Genetics of Ankylosing Spondylitis Consortium AS ImmunoChip study and WTCCC2 controls^1^ were used to localise primary association signals. Haplotypes were inferred using SHAPEIT^21^ with default parameters. Untyped variants were imputed using haplotypes from phase III of the 1000 Genomes Project (October 2014 release)^22^ and using IMPUTE V.2.^23^ Association analysis with a Bayesian logistic model accounting for uncertainty in imputed variants (score method) was performed using SNPTEST.^24^ Population structure was accounted for by including 10 principal components (PCs) as covariates in the regression analysis. Evidence for association is reported as the Bayes factor comparing the model of association with no association. The default priors in SNPTEST for the analysis were used.

### Luciferase reporter assay

The 250 bp sequence flanking *rs4648889* was amplified from human genomic DNA and cloned into the TA cloning kit pCR2.1 vector (Invitrogen, Paisley, UK), then subcloned into the pGL4.23(luc2/minP) reporter vector (Promega, Madison, Wisconsin, USA) at the SacI/XhoI restriction sites (primer sequences available on request). Point mutations corresponding to genetic variants (G/A) of *rs4648889* were introduced using the QuikChange II XL Site-Directed Mutagenesis Kit (Agilent, Santa Clara, California, USA). Luciferase reporter assay details are available online (see online supplementary methods section).

### Patients with AS

Following informed consent (COREC 06/Q1606/139 and OXREC B 07/Q1605/35), venous blood samples were obtained from 19 patients with positive HLA-B27 (average age 51 years, range 29–72) of white British ancestry fulfilling the modified New York criteria for AS.^25^ Sixteen were taking non-steroidal anti-inflammatory analgesics, two were on sulfasalazine but none were currently taking corticosteroids or immunosuppressants. None of the cases had ever received biological therapy, but four with active disease were sampled immediately before starting biological therapy. Twelve cases had low disease activity (<4/10) measured by the Bath AS Disease Activity Index (BASDAI)^26^ but overall there was a considerable range of disease activity (mean BASDAI 4.8/10, range 0.7–10) and C reactive protein (CRP) (mean 17 mg/L, range 0.5–74).

### CD8+ T-cell isolation

CD8+ T cells were isolated from peripheral blood mononuclear cells using the CD8+ T-cell Isolation Kit (Miltenyi, Bisley, UK), according to manufacturer's instructions. CD8+ T cells were then plated for 4 h in RPMI supplemented with 10% fetal bovine serum before harvesting for experiments.

### Electrophoretic mobility gel shift assay

Nuclear extract from Jurkat cells was prepared using the Thermo Scientific NE-PER Nuclear and Cytoplasmic Extraction kit (Thermo Scientific, Waltham, Massachusetts, USA). Electrophoretic mobility gel shift assays (EMSAs) were performed with LightShift Chemiluminescent EMSA Kit (Thermo Scientific, Waltham, Massachusetts, USA) using 5 μg of nuclear extract and 0.6 ng biotin-labelled double-stranded oligonucleotides (50 bp fragment—Eurofins, Wolverhampton, UK). The sequences of the synthetic single-stranded oligonucleotides used in the construction of these double-stranded oligonucleotides are listed in the online supplementary methods.

Probes were prepared using a biotin 3′ end DNA labelling kit (Thermo Scientific, Waltham, Massachusetts, USA).

Single-stranded biotinylated oligonucleotides were mixed and annealed at room temperature for 1 h. Unlabelled competitor probes were used in 100-fold excess.

EMSAs were performed according to standard protocol (Thermo Scientific).

Band intensity was quantified with ImageJ software (Bethesda, Maryland, USA).

A detailed protocol is available online (see online supplementary methods section).

### Chromatin immunoprecipitation—PCR

Chromatin immunoprecipitation (ChIP) was performed using the Diagenode Low Cell# ChIP kit (Liege, Belgium). For each ChIP, 6×10^4^ CD8+ T cells were incubated with 1% formaldehyde for 10 min and 1.25 M glycine added for 5 min. DNA isolation was performed with DNA isolation buffer supplied by the kit. Quantitative PCR (qPCR) was performed on immune complex-associated DNA using allele-specific primers for *rs4648889* (common forward primer: 5′-CCCTACGTGCTTTGCTGTTT-3′, AS risk ‘A’ allele reverse primer: 5′-GGGCCTGGACTCAGGTGT-3′, AS-protective ‘G’ allele reverse primer: 5′-GGGCCTGGACTCAGGTGC-3′), detected with SYBR Green on ABI ViiA7 PCR instruments (Applied Biosystems, Paisley, UK). A compensatory factor ((log(100)/log2) was subtracted from the cycle threshold (Ct) values of the diluted input (1%) in order to calculate the Ct values of the 100% input. Calculation of relative enrichment was done as follows: signals obtained from the ChIP are divided by signals obtained from input sample (representing the amount of the chromatin used in the ChIP)=2^^^ (adj input-ct(IP)).

Relative occupancy was calculated as a ratio of specific signal over background: % input (specific loci)/% input (background loci).

The antibodies used were: IRF4 (Santa Cruz Biotechnology, Dallas, Texas, USA, sc-377383), H3K4Me1 (Diagenode, Liege, Belgium, C15200150) and IgG (Diagenode, Liege, Belgium, C15200001).

### Quantitative real-time PCR

RNA was isolated with TRIzol (Invitrogen, Paisley, UK) and cDNA synthesis (for 500 ng RNA) was prepared with Superscript III from Invitrogen (Paisley, UK). A final concentration of 5 ng/μL was used in qPCR, which was performed with the ABI ViiA7 PCR instrument (Applied Biosystems, Paisley, UK) using SYBR Master mix (Applied Biosystems, Paisley, UK) with evaluation of dissociation curves. mRNA levels of each gene were quantified using the ΔΔCt method and normalised to β-actin. For each gene, PCR melting curves were checked to evaluate the single, specific product.

The specific primers designed were RUNX3 forward: 5′-ACT CAG CAC CAC AAG CCA CT-3′ RUNX3 reverse: 5′-GTC GGA GAA TGG GTT CAG TT-3′ RUNX3 values were normalised to β-actin (Hs_ACTB_1_SG QuantiTect Primer Assay[NM_001101] Qiagen, Manchester, UK).

### Sanger sequencing

Sanger sequencing in forward and reverse orientations was performed by Source Biosciences (Oxford) using the sequencing primer: 5′-GTT TCC ATT CCA CCA ACA CC-3′.

### Statistical analysis

Association data for genotyped and imputed SNPs were obtained on the subset of AS cases of white British ancestry and white British controls from the recent ImmunoChip GWAS. To evaluate the presence of independent effects on genetic susceptibility at the *RUNX3* locus, we performed conditional analysis on 4230 AS cases and 9700 matched controls, as previously described.^1^ Association analysis was performed using the logistic regression function in PLINK (V.1.90),^27^ accounting for population structure with 10 PCs, and conditioning on the *RUNX3* SNP *rs4648889*.

One-way analysis of variance (ANOVA) and two-tailed Student's t test were used to determine statistical significance using the GraphPad Prism software (V.5.03) package.

## Results

### Conditional analysis identifies *rs4648889* as a candidate causal variant

The previous AS ImmunoChip study^1^ identified *rs6600247* as the lead SNP at the *RUNX3* locus (1.3×10^−14^), with *rs4648889* as the next most strongly associated SNP (p=7.6×10^−14^). We performed conditional analysis and found no evidence of independent effects in disease susceptibility between these two SNPs, reflecting the strong LD between them (table 1). After conditioning on SNP *rs4648889*, only two of the other 22 *RUNX3* SNPs previously shown to be strongly associated with AS at p≤10^−11^ retained positive (p<0.02) association—*rs4265380* (1.7×10^−7^) and *rs7529070* (p=1.2×10^−7^). Conditioning on these two SNPs independently, the strong association with the SNP *rs4648889* was retained (p≤3.0×10^−6^ for both SNP), thereby establishing the primacy of this association with AS in this region.

**Table 1:** Conditional analysis of SNP associations at *RUNX3*

Chr.	SNP	Position*	Risk/non-risk allele	Conditional SNP	p Value	OR	RAF (case–control)	LD (r^2^*/*D′) with conditional SNP
1	*rs6600247*	25177701	C/T	*rs4648889*	0.9	1.01	0.54/0.51	0.90/0.97
				*rs4265380*	1.5×10^−4^	1.64		0.97/1
				*rs7529070*	4.9×10^−5^	1.67		0.97/1
1	*rs4648889*	25166416	A/G	*rs6600247*	0.2	0.84	0.54/0.50	0.90/0.97
				*rs4265380*	2.1×10^−6^	3.96		0.94/1
				*rs7529070*	3.0×10^−6^	2.87		0.94/1
1	*rs4265380*	25165943	T/C	*rs6600247*	1.0×10^−6^	0.53	0.55/0.50	0.97/1
				*rs4648889*	1.7×10^−7^	0.22		0.94/1
				*rs7529070*	0.6	1.14		1/1
1	*rs7529070*	25168167	A/G	*rs6600247*	2.1×10^−7^	0.52	0.53/0.50	0.97/1
				*rs4648889*	1.2×10^−7^	0.30		0.94/1
				*rs4265380*	0.3	0.77		1/1

*National Center for Biotechnology Information (NCBI) Build 36 human genome coordinates.

Chr., chromosome; LD, linkage disequilibrium; RAF, risk allele frequency; SNP, single nucleotide polymorphism.

### Identification of a regulatory region upstream of *RUNX3* containing *rs4648889*

**Figure 1:**
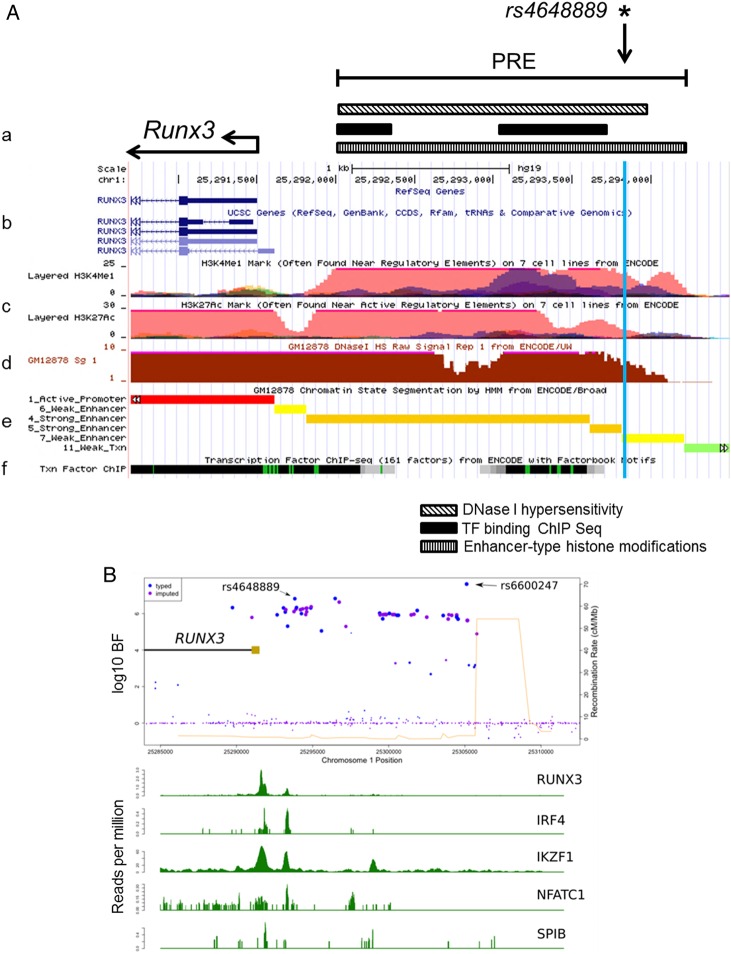
Epigenetic and transcriptional landscape of the 2 kb putative regulatory region containing *rs4648889* upstream of the RUNX3 promoter. (A) Representation of *RUNX3* promoter and putative regulatory element (PRE) location (a). From ENCODE data: *RUNX3* gene location and promoter (b). Histone modification chromatin immunoprecipitation (ChIP)-seq data including H3K4me1 and H3K27Ac (c). DNase I hypersensitivity GM12878 lymphoblastoid cell line (d), predicted chromatin state (e) and condensed transcription factor (TF) ChIP-seq (f) data from various cell lines are shown. (B) *rs4648889* site shows specific TF binding compared with *rs6600247*. ENCODE TF ChIP-seq data from GM12878 lymphoblastoid cell line show differential TF binding between *rs4648889* and *rs6600247* for *RUNX3*, IRF4, IKZF1, NFATC1 and SPIB. Blue and light violet dots represent genotyped and imputed single nucleotide polymorphisms variants and their location; light brown rectangle shows the *RUNX3* promoter.

We first identified a putative regulatory element ∼2 kb upstream of the *RUNX3* distal promoter in the region of *rs4648889* (figure 1A). This was based on published DNase I hypersensitivity sites (DHS) in seven cell types, ChIP-seq peaks for TF binding and the presence of both H3K4Me1 and H3K27Ac histone modifications (figure 1A).^20^ ^28^ This regulatory element also contains a DHS site in CD8+ T cells (http://www.epigenomebrowser.org) and is predicted to bind several TFs. The region around *rs4648889* is more likely to be functionally relevant than *rs6600247*, the lead SNP in the previous ImmunoChip study, considering the paucity of TF binding near the latter (figure 1B). We therefore hypothesised that this region is an active enhancer-like element 5′ of the *RUNX3* promoter (figure 1).

### Differential binding of nuclear extract including IRF4 at *rs4648889*

**Figure 2:**
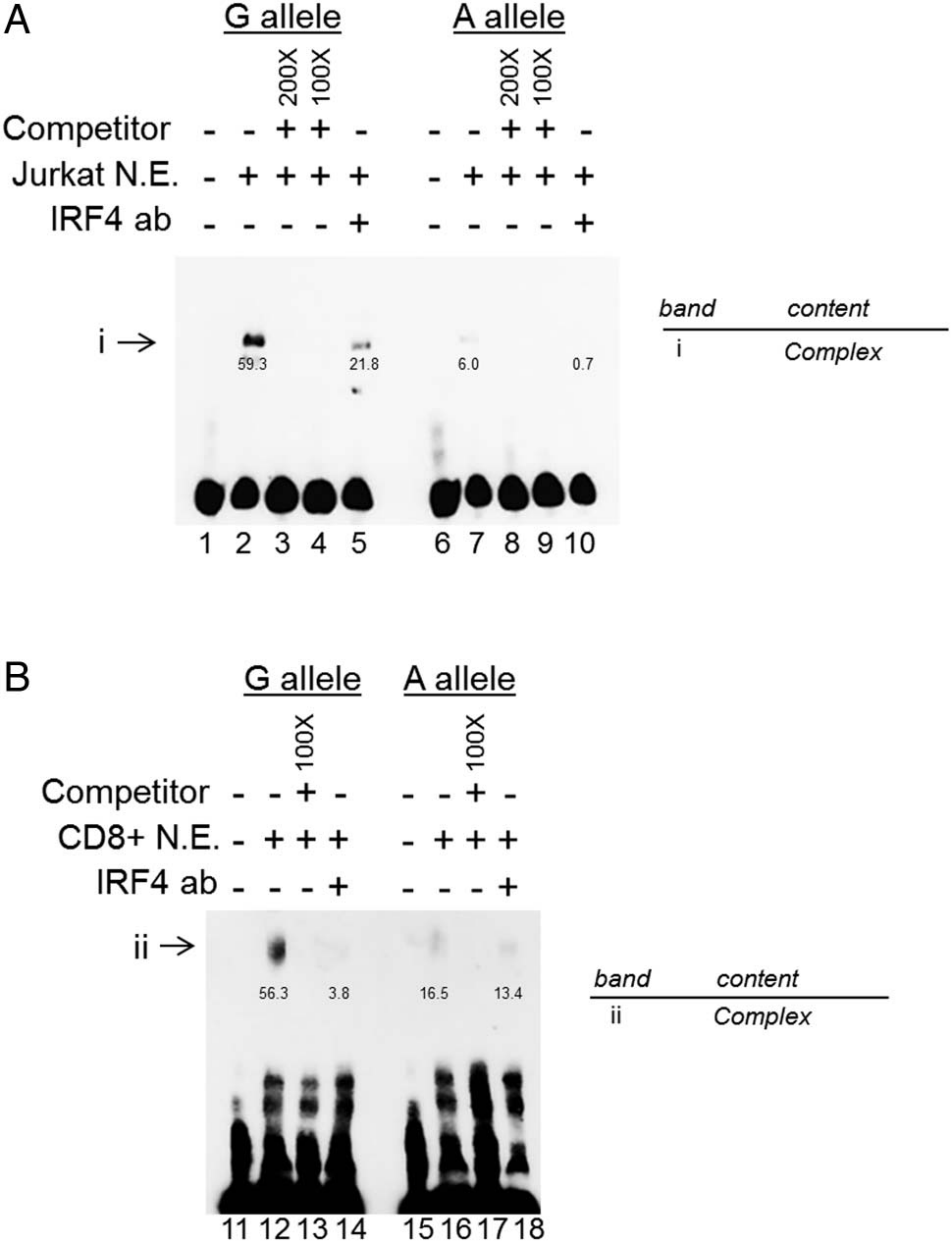
*rs4648889* segment region alters protein–DNA complex formation with evidence of IRF4 involvement. Chemiluminescent electrophoretic mobility gel shift assay (EMSA) (A) showing complex formations (i) after addition of Jurkat nuclear extract (lanes 2 and 7), competition with 200-fold and 100-fold excess of unlabelled probes (lanes 3, 4 and 8, 9) and IRF4 involvement after addition of IRF4 antibody (lanes 5 and 10). IRF4 antibody addition leads to inhibition of the complex (i) (lanes 5 and 10). (B) EMSA showing protein–DNA complex formation (ii) after addition of CD8+ nuclear extract (lanes 12 and 16). IRF4 antibody was used to compete with the labelled oligonucleotides for binding of the nuclear extract (lanes 14–18). Numbers below the bands indicate pixel density. N.E., nuclear extract.

Analysis of ChIP-seq data from the ENCODE project revealed binding of several TFs to the region surrounding *rs4648889* (figure 1). We investigated the effect of the *rs4648889* dimorphism on TF binding to a 50 bp DNA fragment using EMSA. Addition of nuclear extract from Jurkat cells (leukaemia T-cell line) to this DNA probe created a major protein–DNA complex (i) with a visibly weaker band for the AS-risk allele ‘A’ (∼5.4-fold±1.2 SEM less than ‘G’) than the AS-protective ‘G’ allele (figure 2A). Addition of CD8+ T-cell nuclear extract also generated a weaker band for a protein–DNA complex (ii) with the risk ‘A’ allele (∼3.1-fold±1.4 SEM less than ‘G’) compared with the ‘G’ allele (figure 2B). In these experiments Jurkat and CD8+ T-cell nuclear extract binding to the ‘G’ allele was successfully competed by 200-fold and 100-fold excess of unlabelled ‘G’, but not ‘A’ probe and vice versa (figure 2A and see online supplementary figure S1A). The strong binding of both Jurkat and CD8+ T-cell nuclear lysates to the AS-protective ‘G’ allele of *rs4648889* was greatly reduced by adding IRF4 antibody. In contrast, this had little discernible influence on the already very weak binding of either of these nuclear lysates to the AS-risk ‘A’ allele (figure 2A, B).

This suggests that TFs, including IRF4 in the resulting complex, exhibit allele-specific binding to the region around *rs4648889*.

### *rs4648889* alters IRF4 binding and H3K4Me1 histone methylation

**Figure 3:**
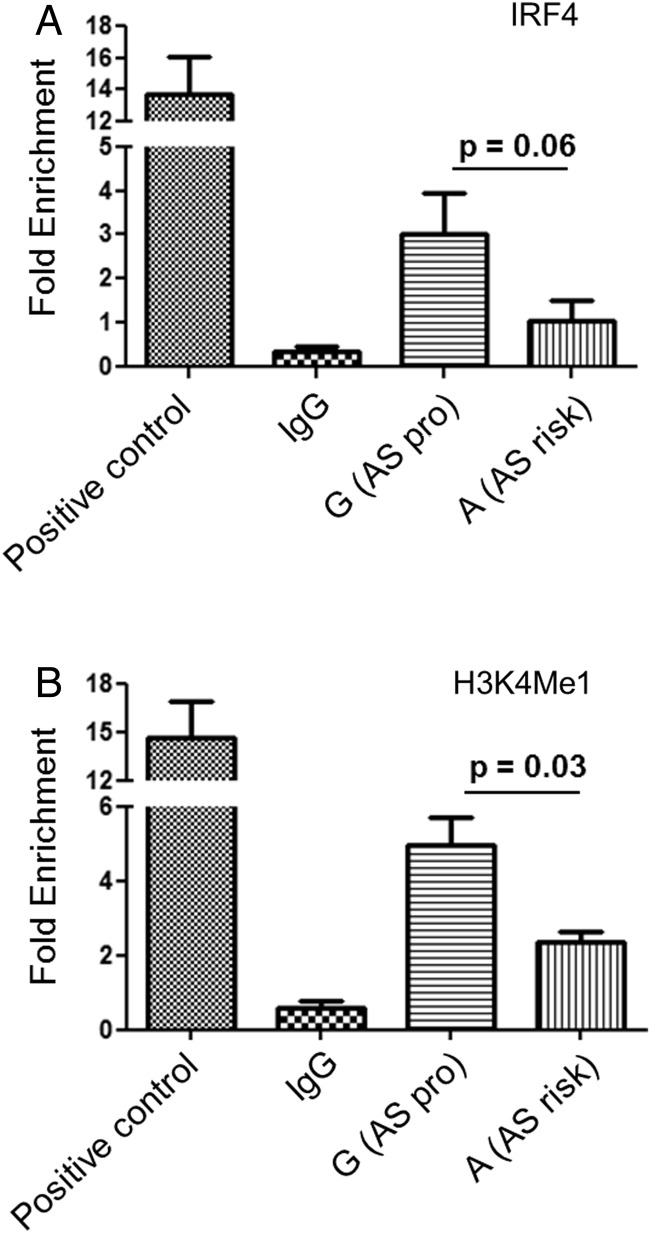
Ex vivo allele-specific binding of IRF4 and H3K4Me1 methylation at the *rs4648889* site. Allele-specific IRF4 (A) and H3K4Me1 (B) chromatin immunoprecipitation (ChIP) quantitative PCR assessed at the *rs4648889* locus in heterozygous CD8+ T cells isolated from three patients with ankylosing spondylitis (AS). The PCR reactions for both the ‘G’ and ‘A’ alleles were done in triplicate in each of the three cases. The relative enrichment is expressed as mean±SEM (p<0.05, Student's t test). Enhancer sequence from *IL10* was used as positive control.

We used ChIP and allele-specific qPCR to assess DNA binding by IRF4 in *rs4648889* heterozygous CD8+ T cells freshly isolated from three patients with AS. Three independent experiments showed IRF4 was preferentially recruited (threefold increase±1.0 SEM, p=0.058) to the AS-protective ‘G’ allele compared with the AS-risk ‘A’ allele (figure 3A).

ChIP-seq data from ENCODE identified the 2 kb region upstream of the *RUNX3* promoter as an enhancer region with increased histone H3K4Me1 in seven different cell lines. We therefore conducted ChIP qPCR for H3K4Me1 on CD8+ T cells. The AS-protective ‘G’ allele had >2.5-fold relative enrichment (p=0.03) compared with the AS-risk ‘A’ allele (figure 3B).

Sanger sequencing of one *rs4648889*-heterozygous AS case showed that the ‘G’ allele was relatively enriched in chromatin fragments immunoprecipitated with antibodies for H3K4Me1 and IRF4 ChIP compared with the reference input (see online supplementary figure S2).

### The AS-risk ‘A’ allele at *rs4648889* shows decreased reporter gene activity

**Figure 4:**
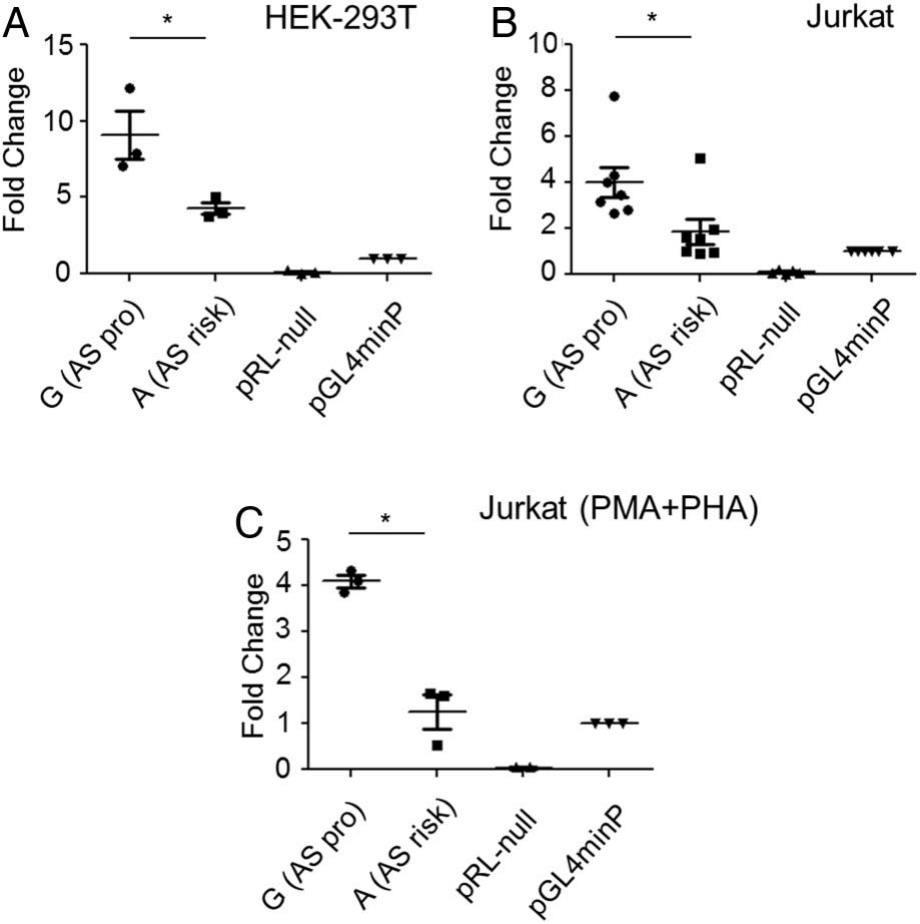
*rs4648889* site shows enhancer activity in vitro. The transcriptional activity of *rs4648889* compared with minimal promoter (minP) was measured by luciferase assays on (A) HEK293T (n=3), (B) Jurkat cells (n=7) and (C) Jurkat cells stimulated with PMA/PHA for 24 h (n=3). The values of relative luciferase activity are expressed as mean±SEM. The relative luciferase activity of the ankylosing spondylitis (AS)-risk ‘A’ allele was significantly reduced compared with the AS-protective ‘G’ allele (*p<0.01, Student's t test).

Reporter activity of the region around *rs4648889* was also evaluated in vitro by luciferase reporter assay. The 250 bp region containing *rs4648889* showed increased luciferase activity compared with the minimal promoter (minP=1) but the presence of the AS-risk allele ‘A’ reduced the enhancer activity from 9.1-fold to 4.3-fold (p≤0.01) in HEK293T cells (human embryonic kidney cell line) and from 4.0-fold to 1.9-fold (p≤0.01) in Jurkat cells (figure 4A, B). The same result was observed in Jurkat cells stimulated with phorbol myristate acetate (PMA)/phytohaemaglutinin (PHA) for 24 h (figure 4C). These findings support the view that the lower enhancer activity at the AS-risk ‘A’ allele is due to decreased H3K4Me1 occupancy.

### The homozygous ‘AA’ genotype at *rs4648889* is associated with reduced *RUNX3* mRNA levels

**Figure 5:**
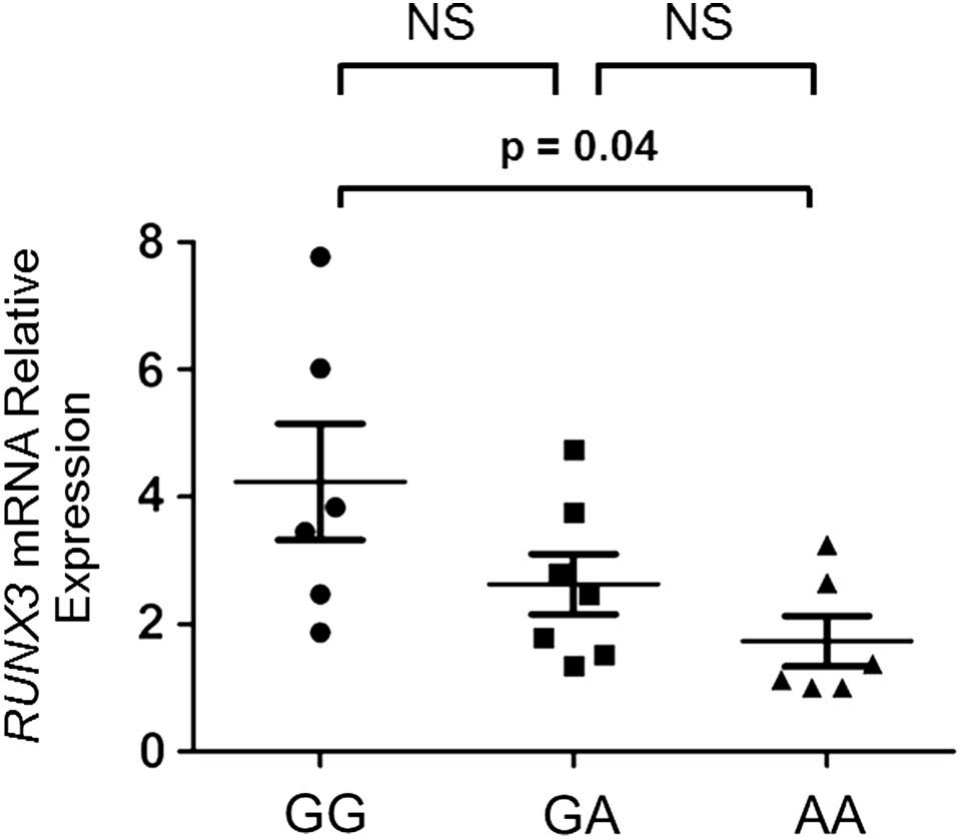
Allele-specific effects of *rs4648889* on RUNX3 expression. Relative amount of *RUNX3* mRNA transcript in primary CD8+ T cells from 19 patients with ankylosing spondylitis having different genotypes was measured by quantitative PCR using the comparative cycle threshold method. The p values were determined using analysis of variance. There was a significant difference (p=0.04) between the GG and the AA homozygotes. The differences between the GA heterozygotes and the GG or AA homozygotes were not significant (NS).

Analysis of *RUNX3* expression (figure 5) in CD8+ T cells isolated from patients with AS having different *rs4648889* genotypes (6 GG, 7 AG and 6 AA) showed that *RUNX3* mRNA levels, normalised against β-actin, were significantly lower in subjects with the AA than the GG genotype (1.7±0.9 vs 4.2±2.2 SEM, ANOVA p≤0.05). No correlation was observed between *RUNX3* expression and disease activity as defined either by BASDAI or CRP.

## Discussion

We have shown here that *rs4648889* is probably the primary AS-associated SNP at the *RUNX3* locus and that this association can probably be best explained through its effects on gene expression. The *rs4648889* AS-risk allele ‘A’ exhibits relatively low luciferase reporter activity compared with the AS-protective ‘G’ allele, less *RUNX3* mRNA levels and less enrichment for H3K4Me1 histone modification of a type associated with active enhancer elements.^7^ The AS-risk allele ‘A’ is also reproducibly associated with reduced formation of protein–DNA complexes that contain IRF4. The TF IRF4 is involved in regulating the number and function of CD8+ T cells,^29–32^ influencing their differentiation, expansion^30^ ^33^ and metabolism.^31^ Our results are consistent with previous reports that selective IRF4 binding to the distal promoter exerts an inhibitory effect on *RUNX3* expression and consequently on CD8+ T-cell development and function.^32^ Previous studies have indicated that the total number of peripheral blood CD8+ T cells is significantly decreased in AS cases, and that this is strongly correlated with the *RUNX3* genotype.^1^ ^2^ We believe that this may be mediated through differential allelic binding of TF complexes (including IRF4) to this *RUNX3* enhancer. *RUNX3* plays a key role in haematopoiesis and enhances CD8 T-cell development/activation and has an inhibitory effect on CD4+ T cells.^9^ ^10^ *RUNX3* affects NK cell development,^10^ mediates transforming growth factor-β responses in the activation of dendritic cells during inflammation^34^ and, together with T-bet, regulates interferon-γ and interleukin 4 expression in T-helper (Th)1 cells.^35^

The striking HLA-B*27 association with AS has long tempted speculation that aberrant immune responses to microbial infection, perhaps mediated through *HLA-B27*-restricted CD8+ cytotoxic T cells, could be involved.^36^ ^37^ Such ideas have been strengthened by the observation that HLA-B*27-transgenic rats develop SpA unless reared in germ-free conditions.^38^ However, HLA-B*27 transgenic rats still develop SpA in the absence of CD8+ T cells.^39^ Our results would be consistent with the hypothesis that reduced CD8+ T-cell numbers and/or function play a role in the pathogenesis of AS. More than 40 genetic influences have been identified in AS, several of which (HLA-B*27, *RUNX3*, *EOMES*, *TBX21*, *ZMIZ1*, *IL7* and *IL7R*) potentially affect lymphocyte biology.^1^ Currently, the full complexity of immune cell involvement in AS is incompletely understood. Here we have demonstrated potential epigenetic regulatory effects at the *RUNX3* locus in CD8+ T cells. We have not yet extended these studies to other cell types, such as Th1, NK, dendritic or other immune cells that could also be involved in AS.^40^ For example, reduced *RUNX3* expression could enhance CD4+ T-cell activity, which would be consistent with models of AS invoking a pathological role for CD4+ T cells.^41^ ^42^ In particular, CD4+ Th17 cells have been implicated in AS and appear to be present in increased numbers in the peripheral blood of patients with pre-radiographical axial SpA^43^ and reactive arthritis.^44^

It is likely that most genetic associations in AS reflect relatively subtle changes in gene expression. The differences in DNA–protein complexes revealed by EMSA experiments between the AS-risk ‘A’ and AS-protective ‘G’ allele require further investigation to define the full diversity of TFs involved in these complexes and also the network of genes that might ultimately be regulated by the *rs4648889* polymorphism. It is well known that eukaryotic transcription may be controlled by regulatory elements that are distant from their target genes; such control can be exerted (sometimes on multiple genes) through the formation of chromatin loops.^45^ ^46^ We have not formally excluded the possibility that this *RUNX3* regulatory region could influence other more distant genes relevant to AS. Establishing the full extent of the genes (and their regulation) in these pathways and their relationship to the pathogenesis of AS represents a considerable challenge. In future we will investigate TF binding and chromatin accessibility across the whole genome to explore the composition of these AS gene regulatory networks.^47^

This work illustrates the first steps towards a more complete mechanistic understanding of just one of the many genetic associations of AS. These and related techniques have broad applications to the investigation of other similar regulatory polymorphisms that probably account for much of the genetic risk in AS. A deeper knowledge of the factors involved in these processes is likely to lead of the discovery of new therapeutic targets.

## Supplementary Material

Web supplement
