# Supplementary material for: The genetic association of RUNX3 with ankylosing spondylitis can be explained by allele-specific effects on IRF4 recruitment that alter gene expression
Source: Ann Rheum Dis. 2015 Oct 9;75(8):1534–40. doi: 10.1136/annrheumdis-2015-207490 (PMC4975853; doi:10.1136/annrheumdis-2015-207490)

# Supplementary Methods

The genetic association of *RUNX3* with ankylosing spondylitis can be explained by allele-specific effects on IRF4 recruitment that alter levels of gene expression

Matteo Vecellio[1,2,3], Amity R Roberts[1,2,3], Carla J Cohen[1,2,3], Adrian Cortes[4,5], Julian C Knight[5], Paul Bowness[1,2,3] and B Paul Wordsworth[1,2,3]

**Author affiliations**

[1]Nuffield Department of Orthopaedics, Rheumatology and Musculoskeletal Sciences, University of Oxford, Oxford, UK

[2] National Institute for Health Research Oxford Musculoskeletal Biomedical Research Unit, Oxford, UK

[3] National Institute for Health Research Oxford Comprehensive Biomedical Research Centre, Botnar Research Centre, Nuffield Orthopaedic Centre, Oxford, UK

[4]Nuffield Department of Clinical Neurosciences, Division of Clinical Neurology, John Radcliffe Hospital, University of Oxford, Oxford, UK

[5]Wellcome Trust Centre for Human Genetics, Roosevelt Drive, University of Oxford, Oxford, UK

**Address for correspondence:**

Prof BP Wordsworth

NIHR Oxford Musculoskeletal Biomedical Research Unit,

Botnar Research Centre,

Nuffield Orthopaedic Centre

Windmill Road,

Headington,

Oxford

OX3 7LD

Email: paul.wordsworth@ndorms.ox.ac.uk

Telephone: 44 (0)1865741155

### Luciferase Reporter Assay

In details, HEK293T and Jurkat cells were cultured in DMEM and RPMI, respectively, supplemented with 10% fetal bovine serum (FBS), 100 units/ml penicillin/ streptomycin and 2 mM L-glutamine. 200,000 HEK293T or Jurkat cells/well in 96/24-well plates, were co-transfected with 100/500 ng of pGL4 construct and 2/10 ng of pRL-null (Promega, Madison, USA) using GeneJuice (Novagen, Darmstadt, Germany) and GeneIN™ (GlobalStem, Gaithersburg, USA) respectively. After 24 hours transfection, Jurkat cells were stimulated with PMA/PHA (1 µg/ml PHA + 50 ng/ml PMA, Sigma Aldrich, Gillingham, UK) for 16 hours. After 48 hours in total, luciferase activity was measured using the Dual-Luciferase assay reporter system (Promega, Madison, USA). Firefly luciferase activity was normalized relative to Renilla luciferase activity for each transfection and calculated as fold increase over pGL4.23[luc2/minP].

Student *t*-test and one-way ANOVA were used to determine significant differences between the two allelic constructs.

### Electrophoretic Mobility Gel Shift Assay (EMSA)

Briefly, EMSA were performed as follows: incubations were done at room temperature for 40 minutes in total. IRF4 Antibody (Santa Cruz Biotechnology, Dallas, USA sc-377383, 5 µg) was added to the nuclear extract (Jurkat or CD8+ T-cells) for 20 minutes, followed by DNA for additional 20 min. Reactions were run chilled on pre-cast non-denaturing 6% DNA retardation gels (Invitrogen, Paisley, UK) with 0.5-fold TBE running buffer (45 mM Tris, 45 mM boric acid, 1 mM EDTA, pH 8.0).

DNA was then transferred at 380 mA from the gel to a pre-activated nitrocellulose membrane for 30 to 60 minutes on ice and cross-linked by UV. All blocking and detection incubations were performed according to manufacturer's instructions (Thermo Scientific, Waltham, USA) and the membrane was exposed to X-ray film.

The sequence of the DNA probes are:

*rs4648889* Forward, A allele: 5'-CCT GAG GGG CTT CCC CCT CCC TGG A*AA CCT GAG TCC AGG CCC AGG AAG G-3',

*rs4648889* Reverse, A allele: 5'-CCT TCC TGG GGC CTG GAC TCA GGT TT*C CAG GGA GGG GGA ACC CCT CAG G-3';

*rs4648889* Forward, G allele: 5'- CCT GAG GGG CTT CCC CCT CCC TGG G*AA CCT GAG TCC AGG CCC AGG AAG G-3',

*rs4648889* Reverse, G allele: 5'- CCT TCC TGG GGC CTG GAC TCA GGT TC*C CAG GGA GGG GGA ACC CCT CAG G -3'.

The stars highlight the *rs4648889* position.

**Suppl. figure 1:** ***rs4648889*** **segment region alters protein/DNA complex formation confirming IRF4 involvement and specificity.** (A) Representative chemiluminescent EMSA showing competition experiments with 100 fold excess of unlabelled competitor for both alleles (lanes 3, 4, 7 and 8). (B) Effect of nonspecific IgG antibody addiction (lanes 11 and 14). (C) Representative EMSA showing binding for IRF4 purified protein, with preference for the AS-protective allele “G” (lanes 17 and 18).

**Suppl. figure 2: Sanger sequencing after ChIP-qPCR of H3K4Me1 and IRF4.** ChIP followed by sequencing for the *rs4648889*-containing region in heterozygous CD8+ T-cells from AS patients. Chromatograms represent one ChIP experiment generated and sequenced. Levels of H3K4Me1 and IRF4 are relatively higher for the protective “G” allele, compared to the risk “A” allele, after normalization on input.

**Suppl. figure 3: IRF4 protein expression in cell lines used in reporter assay and EMSA.** (A) Western blot analysis showing IRF4 protein expression: 51 kDa band was detected in HEK 293T, Jurkat and Raji cell lines. Band density is reported in the associated bar graph (n= 3 for each group). (B) Real-Time PCR showing IRF4 mRNA expression in HEK 293T, Jurkat and Raji cell lines (n=2, in triplicate).

# Supplementary Figure 1

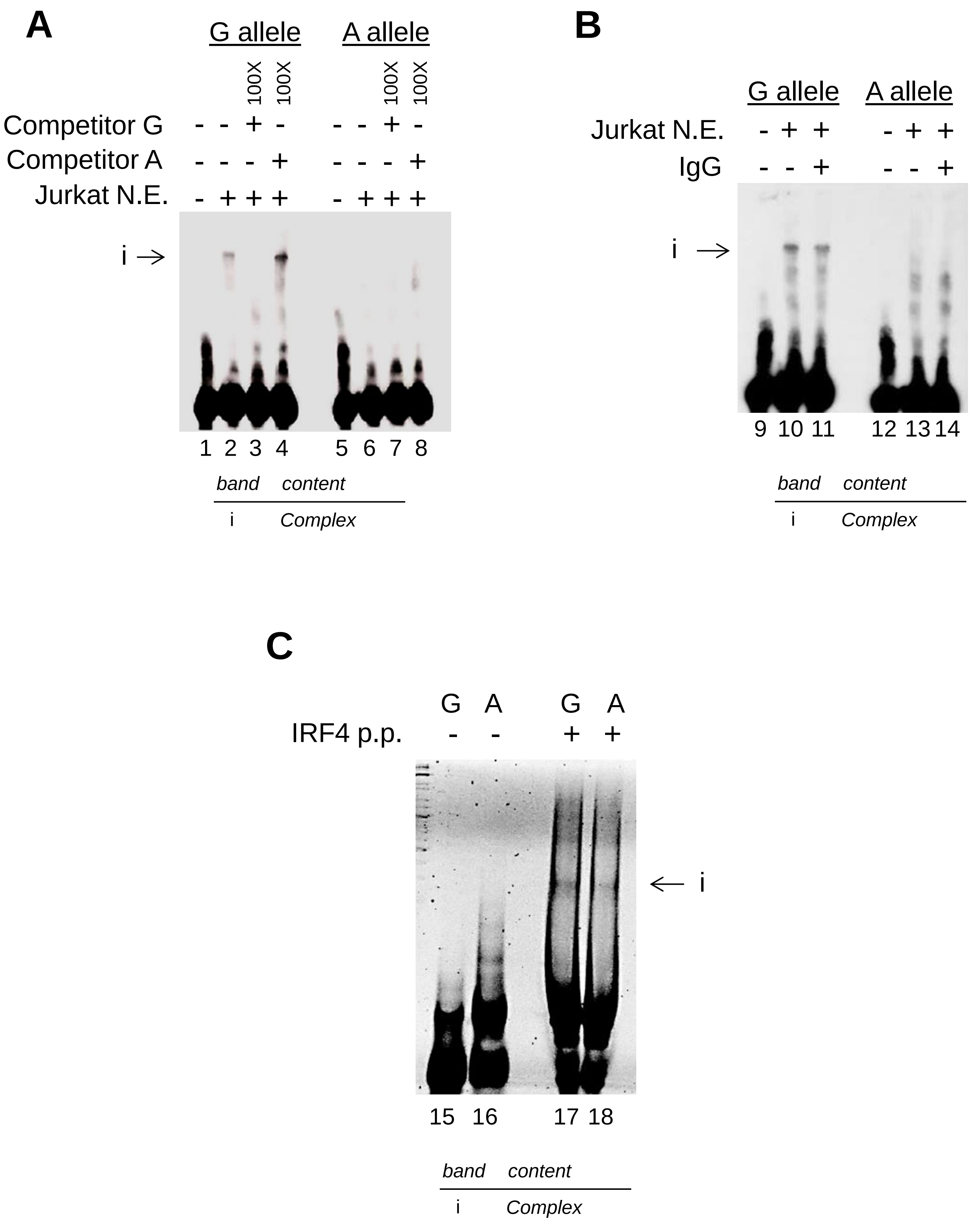

# Supplementary Figure 2

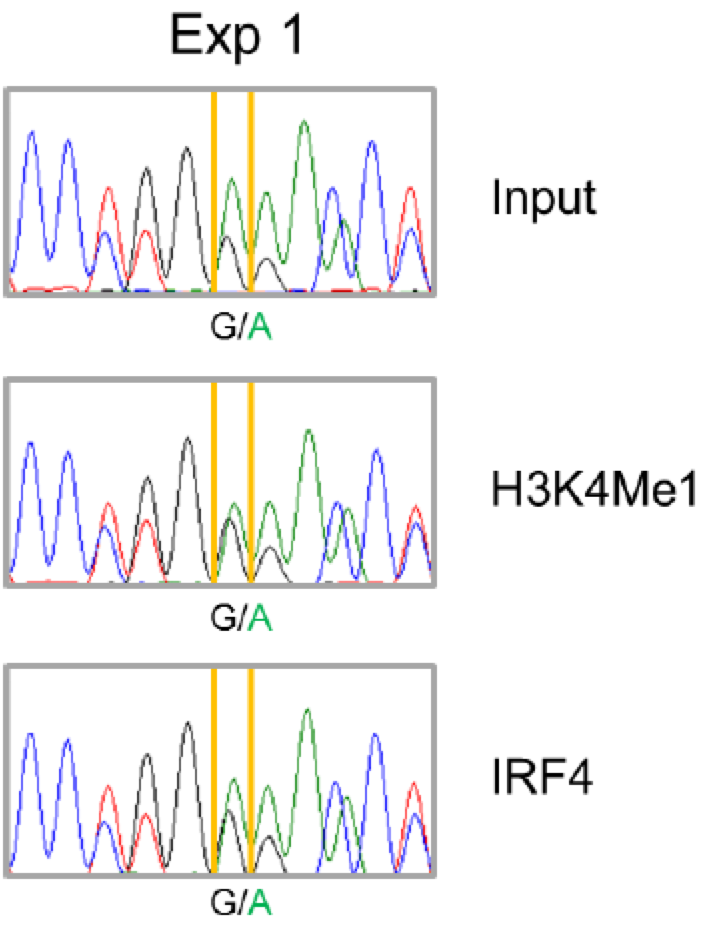

# Supplementary Figure 3

**A**

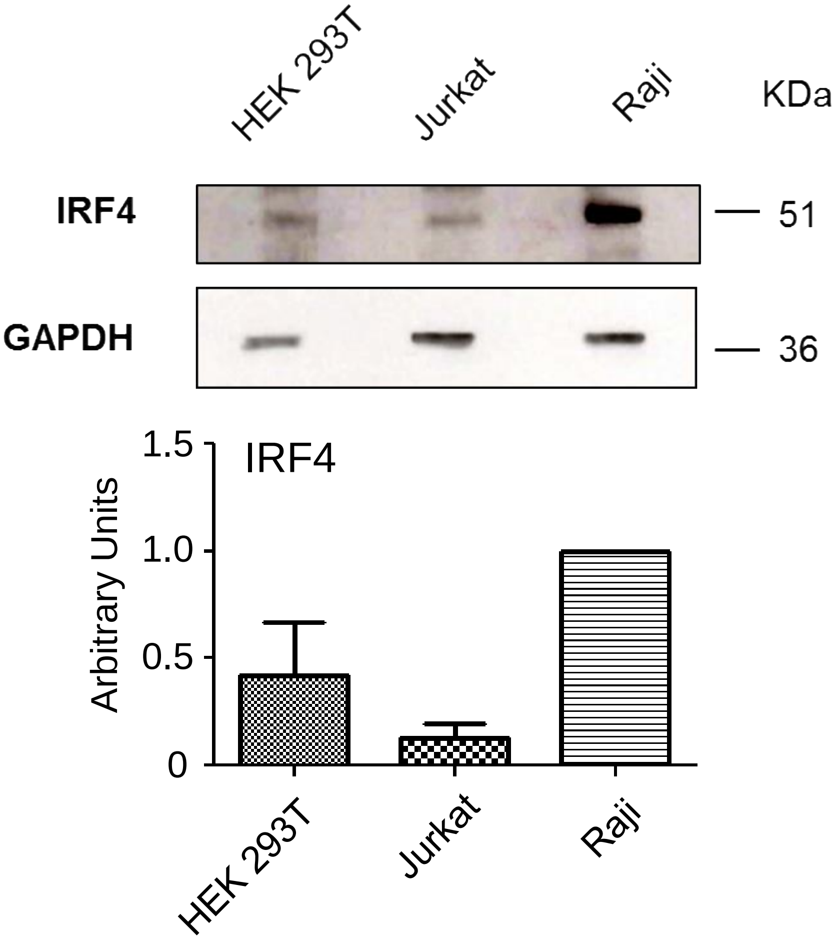


**B**

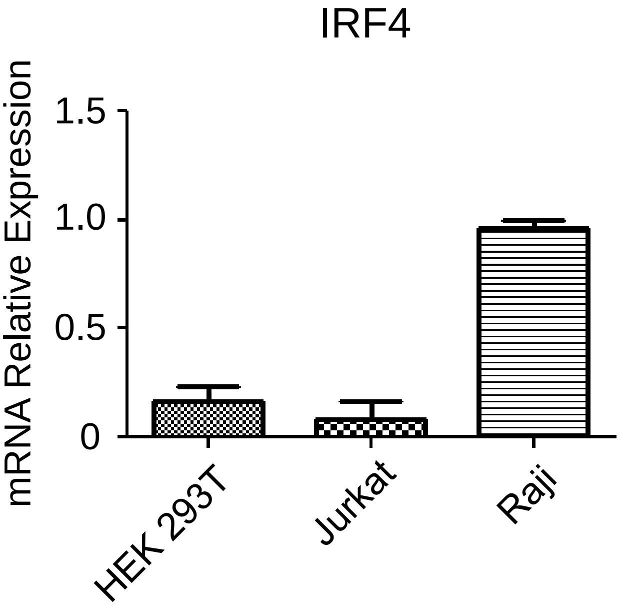

Supplement: Web supplement [file annrheumdis-2015-207490-s1.pdf]
